# Glandular Fever Testing in Patients Presenting With Tonsillitis: A Retrospective Study

**DOI:** 10.7759/cureus.50213

**Published:** 2023-12-09

**Authors:** Abubaker Elamin, Amena Al Saad, Laith Sinan, Ahmed Bayoumi, Abdelrahman Ezzat Ibrahim

**Affiliations:** 1 General Surgery, Humanitas University, Milan, ITA; 2 Otolaryngology, Nottingham University Hospitals, Nottingham, GBR; 3 Otolaryngology, United Lincolnshire Hospitals, Lincoln, GBR; 4 General Surgery, Nottingham University Hospitals, Nottingham, GBR; 5 Orthopaedic Surgery, Nottingham University Hospitals, Nottingham, GBR; 6 Otolaryngology - Head and Neck Surgery, United Lincolnshire Hospitals, Lincoln, GBR

**Keywords:** adult tonsils, epstein- barr virus, ebv, glandular fever, tonsillitis

## Abstract

Background: Tonsillitis, the inflammation of the palatine tonsils, typically arises from infections and predominantly affects children of primary school age. Most cases stem from viral or bacterial infections. Glandular fever, a subtype, is primarily caused by the Epstein-Barr virus (EBV). This specific type of tonsillitis can lead to severe complications, including splenic rupture, encephalitis, chronic fatigue, and acute acalculous cholecystitis. As a result, early identification is vital to establish proper safety measures and prevent the onset of these potentially dangerous complications in patients.

Objectives: This study aims to determine the number of missed glandular fever cases diagnosed as simple tonsillitis and raises considerations in managing these patients.

Methods: A 12-month retrospective single-centre cohort study was carried out in Lincoln County Hospital, Lincoln, England. A total of 185 patients diagnosed with tonsillitis were included, along with their presenting symptoms and investigations, including liver blood tests and glandular fever screening.

Results: Among the 185 patients, averaging 26 years old, 112 were screened for EBV infection, revealing 35 positive cases (31.3%). Notably, 74% of these positive cases (26 out of 35) displayed abnormal liver function test results.

Conclusion: Applying the percentage of EBV-positive cases to the 73 unscreened patients results in a likelihood of 23 missed cases of EBV infection. These form 12% of the study group, indicating a significant potential missed number of cases. Given the associated risks and complications with EBV, we note the importance of screening to identify cases and apply relevant considerations in their management.

## Introduction

Definition

Sore throat is a widespread presentation among patients in the United Kingdom, and tonsillitis, whether complicated or simple, is often the underlying cause [1]. Tonsillitis is the inflammation of the tonsils' parenchyma, which is a lymphatic tissue located in the pharynx. Most infected are the palatine tonsils, which are two glands situated between the palatoglossal arch and the palatopharyngeal arch, specifically at the isthmus of the fauces. Anatomically, the palatine tonsils form a part of Waldeyer's ring, which also involves the pharyngeal, tubal, and lingual tonsils [2]. Alongside a sore throat, patients with tonsillitis can present with glandular swelling, fever, difficulty swallowing, changes in voice, headache, and ear pain [3].

Tonsillitis can be subdivided further into acute, recurrent, and chronic tonsillitis. Acute tonsillitis is defined as an infection lasting between four and 14 days, whereas recurrent tonsillitis is several episodes of acute tonsillitis over the year. Chronic tonsillitis is diagnosed when a persistent infection of the tonsils causes long-term inflammation [4]. Tonsillitis infection can be associated with infectious mononucleosis or glandular fever, a clinical syndrome presenting with the classic triad of fever, pharyngitis, and lymphadenopathy. Glandular fever can also present with signs of splenomegaly, hepatomegaly, myalgia, and jaundice. Complications include splenic rupture, encephalitis, autoimmune diseases, chronic fatigue, acute acalculous cholecystitis, lymphoma, and other malignancies [5]. Therefore, it is essential to identify patients early on to recognise risks and provide adequate safety netting.

Aetiology and risk factors

Tonsillitis is usually infectious in nature and occurs mostly due to viral infections such as Epstein-Barr virus (EBV), adenoviruses, respiratory syncytial virus (RSV), and influenza, but can also be bacterial due to B-haemolytic *streptococcus*, mycoplasma, and *Corynebacterium diphtheriae* [6]. In glandular fever tonsillitis, EBV is the leading cause. However, it can be caused by other organisms such as cytomegalovirus (CMV), human herpesvirus 6 (HHV6), herpes simplex virus (HSV), *Streptococcus pyogenes*, *Toxoplasma gondii*, and HIV-1 [7]. Epstein-Barr virus is a double-stranded DNA herpes virus that is often transmitted through bodily fluids, such as saliva. Therefore, deep kissing and sharing food can spread the infection. It can also spread via semen during sexual contact and blood through blood transfusion, as well as stem cell and organ transplants [8]. Once contracted, EBV can remain latent in the body without symptoms, colonising mainly in the tonsils over time. The incubation period, ranging from four to seven weeks, is contagious but can remain so after the infection. There is no current evidence of gender or seasonal factors in the incidence of glandular fever [9].

Epidemiology: incidence and prevalence

In the United Kingdom, around 550 consultations in an average-sized general practice are due to a sore throat. In a year, there are approximately 100 per 1,000 people with a recurrent sore throat incidence [10]. Epstein-Barr virus is a ubiquitous virus that has infected around 95% of young adults worldwide. Epstein-Barr virus' seroprevalence increases with age, with 93% of seropositive cases seen in ages 22-24. Thus, glandular fever incidence has shown a peak incidence between the ages of 15 and 24 [11]. Specifically, in England, a study has shown there is steady seroprevalence up to the age of 11, increasing slightly in the ages 12-18 to around 70%, and the highest prevalence rate of around 90% for those aged 19 and over [12].

In glandular fever tonsillitis, EBV was shown to have been the causative organism in 90% of adults [13]. Several studies have investigated the association rate between EBV and tonsillitis, and most have proven a high correlation. A study held in the United Arab Emirates has shown that 43% of tonsillectomy specimens had EBV infection, while another study in the Mexican population has shown a 46% viral detection [14, 15]. Additional research on tonsil specimens has found the EBV genome in 54% of the samples, suggesting that tonsils act as a reservoir for EBV, causing recurrent tonsillitis [16]. Given the high association and prevalence, it is crucial to consider glandular fever screening in tonsillitis patients.

As previously mentioned, EBV can lead to several malignancies, including nasopharyngeal carcinoma (NPC), gastric carcinoma (GC), Hodgkin lymphoma (HL), Burkitt lymphoma (BL), and a few others [17]. It was also noted that the prevalence of EBV-related malignancies has been increasing over the years, affecting approximately 1% of the world population [18]. Furthermore, infectious mononucleosis caused by EBV is considered a risk factor for developing Hodgkin's disease [19]. According to an international comparative study in 2017, EBV constituted 18% of the incidence and 17% of mortality in the malignancies mentioned earlier. It also showed that EBV-attributed malignancy incidence and mortality increased by 36% and 19%, respectively, from 1990 to 2017 [20]. This provides further evidence of the impact of EBV infection and elicits the need to screen suspected patients to provide appropriate management.

Investigations and management

Tonsillitis can progress to form a peritonsillar abscess or quinsy; hence, it is important to ensure there is no airway compromise and check for sepsis. Examination findings of lymphadenopathy and splenomegaly, along with the history to assess for risk factors, increase the likelihood of infectious mononucleosis [21]. With tonsillitis, a full workup blood test is usually carried out to examine the infection markers (C-reactive protein (CRP), white blood cells (WBC), and lymphocytes), liver and kidney functions, blood cultures, and glandular fever screen [22]. With glandular fever screening, the heterophile antibody or monospot test has low sensitivity and a high rate of false negatives, which is no longer recommended by the Centres for Disease Control (CDC), but EBV serology testing has been the most reliable [23, 24]. As mentioned before, most of the population has acquired EBV and is seropositive. However, serology testing can reveal if there's an acute infection via three antibodies [24]: (1) viral capsid antigen (VCA) IgM antibodies, which indicate early infection stages and last four to six weeks; (2) VCA IgG antibodies, which peak two weeks after acute infection, then drop and persist for life; (3) anti-EB nuclear antigen (EBNA), which indicates previous infection and is usually detected after two to four months, persisting for life.

The management of tonsillitis will depend on clinical assessment and investigation results. Within primary care, National Institute for Health and Care Excellence (NICE) guidelines advise using the FeverPAIN (an acronym for fever last 24 hours, pus on tonsils) score or Centor score to determine the possibility of group A beta-haemolytic *Streptococcus *(GABHS) infection and, thus, the need for antibiotics. Hospital admission is considered when signs of dysphagia or airway compromise are present, as well as complications such as quinsy [25]. Where EBV is suspected or confirmed, the infection is usually self-limiting. The mainstay of therapy is supportive care, including good hydration, antipyretics, and analgesics, while steroids are not routinely administered unless there is a risk of airway compromise [21, 26]. Epstein-Barr virus patients are advised to refrain from strenuous physical activity and contact sports in the initial three to four weeks (up to eight weeks in some cases) of illness due to the increased risk for splenic rupture [21, 27]. Despite the associated risks and complications, no current ENT guidelines advise which patients should be screened for glandular fever. In our research, we aim to assess the number of potential missed EBV cases in those presenting with tonsillitis and thus identify the risk of developing EBV-related disorders.

## Materials and methods

A retrospective single-centre cohort study was conducted at Lincoln County Hospital in Lincoln, England, and focused on patients exhibiting symptoms of tonsillitis over a 12-month period, spanning from May 2022 to April 2023. The study methodology involved meticulously examining daily updated handover documents within the ENT department. These documents encompassed details concerning referrals and admissions, aiding in the identification of patients presenting with tonsillitis symptoms during the specified timeframe.

Following the identification process with a total of 185 patients, a thorough investigation was undertaken into each patient's initial symptoms and diagnostic evaluations. This comprehensive analysis included an assessment of presenting symptoms along with a review of various medical examinations, such as blood tests and specific screenings for glandular fever. Patients were stratified based on whether they received screening for glandular fever or not, and subsequent results were meticulously recorded, documenting both positive and negative outcomes.

Utilising the probability principle, which states that in a finite non-empty sample space with equally probable outcomes, the probability of an event contained within the sample space is determined by the ratio of outcomes in that event to the total outcomes in the sample space, we assessed the probability of undetected cases of glandular fever among tonsillitis patients who were not tested.

Moreover, the study placed emphasis on evaluating liver function abnormalities among all tonsillitis patients to elucidate potential correlations with EBV infection, a common cause of glandular fever. The intent was to explore any potential associations between glandular fever, liver function derangements, and tonsillitis presentations within this patient cohort.

## Results

Demographics

**Figure 1:**
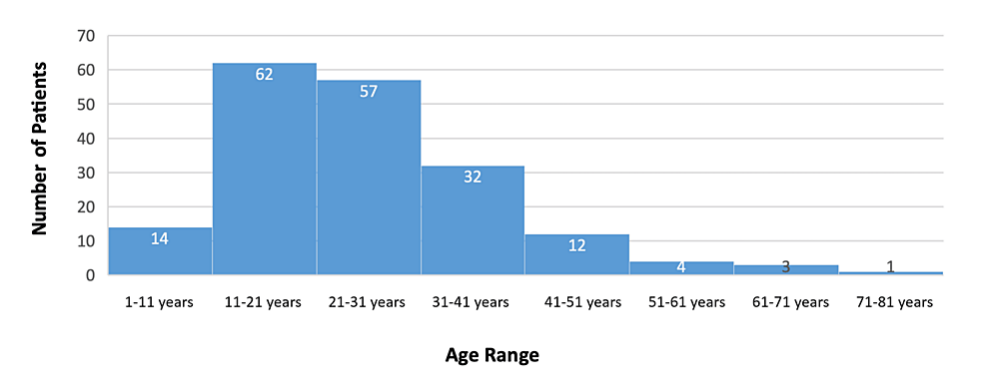
The distribution of participants as per their age range. The data have been represented as N.

Our research gathered data from 185 tonsillitis patients over a period of 12 months. The age range for this cohort was from one to 80 years, with the average age being approximately 26 years. When examining the age distribution of those patients, 62 patients fell into the 11-21 age range, while 57 patients fell into the 21-31 age range. This shows that over 60% of our study participants were young adults (Figure 1).

**Figure 2:**
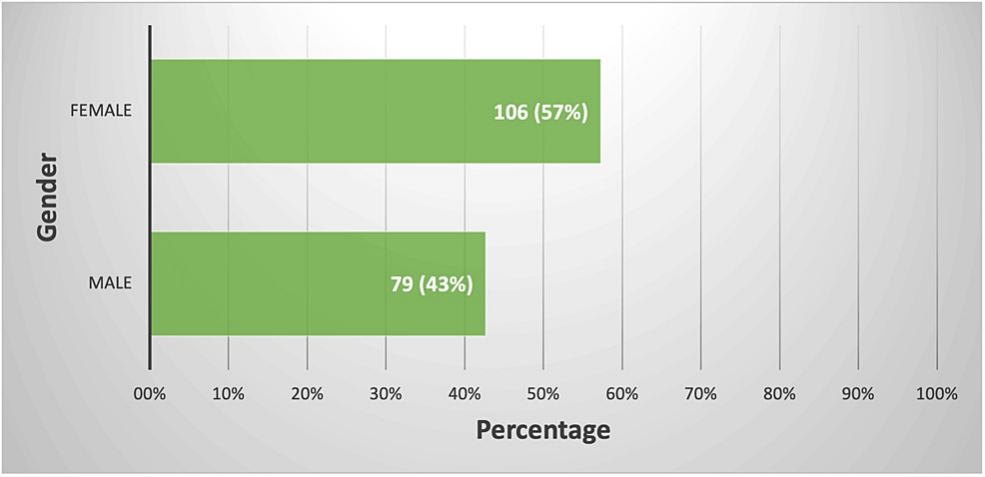
The distribution of participants as per their gender. The data have been represented as N (%).

Regarding gender, approximately 106 (57%) of the cohort were females, whereas 79 (43%) were males (Figure 2).

Investigations

**Figure 3:**
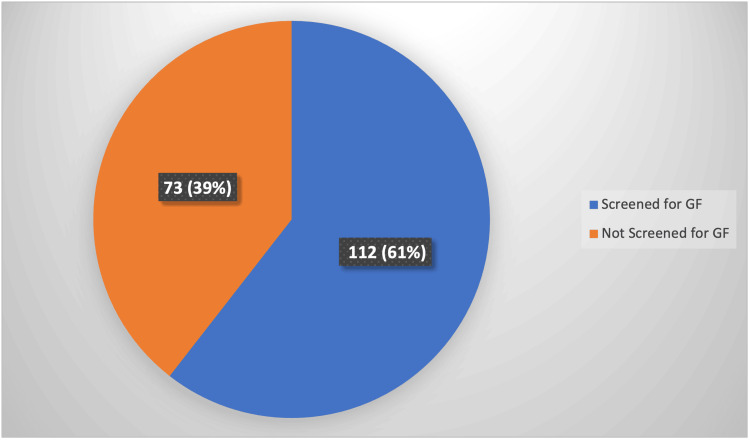
Screening rate for glandular fever (GF) The data have been represented as N (%).

Out of the 185 patients presenting with tonsillitis between May 2022 and April 2023, we found that 112 (61%) patients had undergone a glandular fever screen test, representing approximately two-thirds of the cohort and leaving 73 (39%) untested (Figure 3).

**Figure 4:**
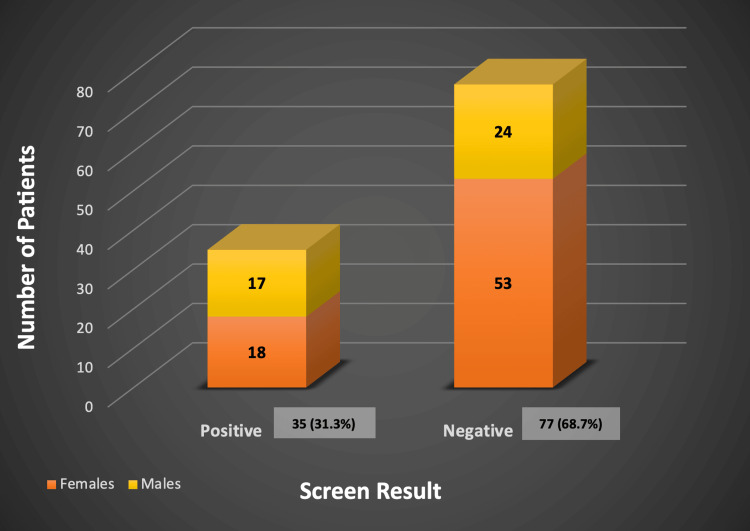
Screening results for glandular fever The data have been represented as N (%).

Among the 112 (61%) patients who had been tested, results showed that 77 (68.7%) patients tested negative, and 35 (31.3%) patients tested positive. Patients with negative results included 53 females and 24 males, while those with positive screen results included 17 males and 18 females (Figure 4).

**Figure 5:**
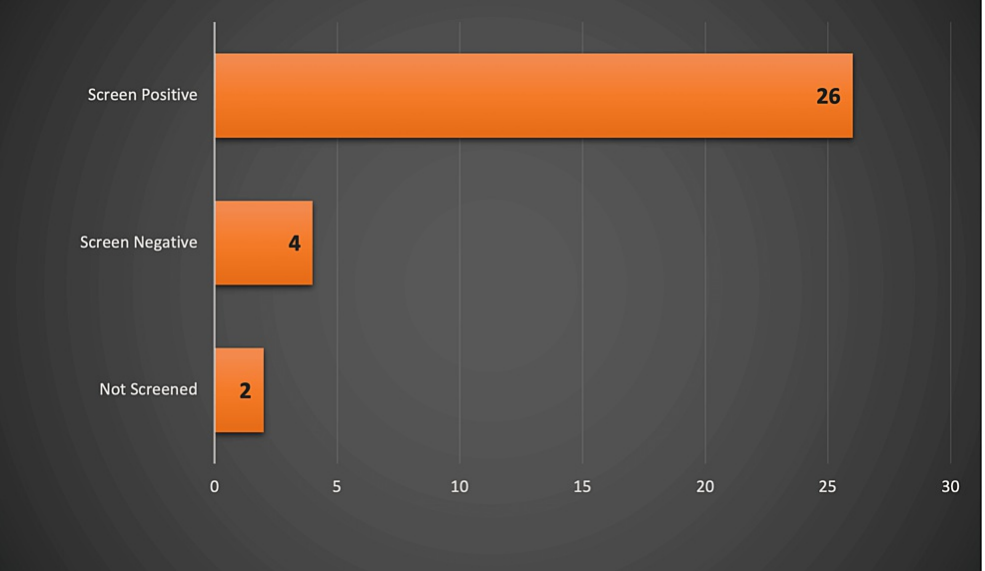
Patients with deranged liver function tests in relation to glandular fever screening results The data have been represented as N.

Looking at the age prominence in the 35 positive cases with EBV infection, the average age was around 21 years, and 30 (85%) patients were found to be over 16 years of age. From another perspective, 32 out of the 185 tonsillitis patients were found to have deranged liver function tests on their blood tests. Upon closer inspection of the 32 patients, 26 were EBV-positive, four were EBV-negative, and two were not screened (Figure 5).

This shows that 26 (74%) of the 35 patients with EBV infection had deranged liver function tests, indicating the adverse effects of the infection.

## Discussion

As previously mentioned, among the tonsillitis patients, we found 112 patients were tested for glandular fever, while the remaining 73 patients were not. Consequently, when looking at the results of the 112 tested patients, we found that 35 (31%) tested positive. If we look at this from a different perspective, we can use this percentage of positive results as a guide to help determine how many possible positive cases are missed. In other words, when we look at the 73 untested patients from the cohort and apply the possibility of 31% of them being positive, we get approximately 23 patients. This means that 23 patients from the non-screened group are possibly 'missed' EBV-positive cases, who would have been managed without considering the risks and complications associated with the infection.

**Figure 6:**
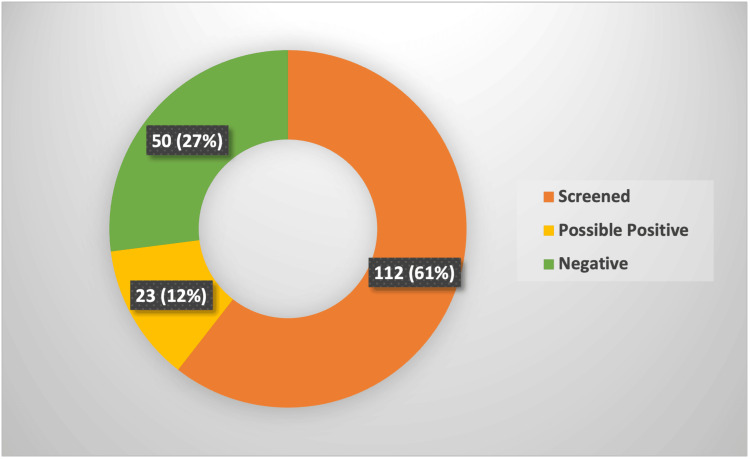
Possible missed Epstein-Barr virus cases The data have been represented as N (%).

Those 23 patients form 12% of the study group (Figure 6).

This highlights the fact that such a significant number was found in a 12-month study in a relatively small city. If we were to investigate the potential missed cases in the whole county, let alone the country, the number would be significantly higher. With no current guidelines regarding when screening for EBV is indicated, there is a higher risk of missing EBV cases and the consequences of mismanagement. It is worth noting the impact of national guidelines. Therefore, a consensus advising on the investigations, including EBV screening, of such cases should be in place.

A high percentage of EBV-positive cases showed an abnormal status of their liver function, suggesting adverse consequences of the EBV infection. Due to the substantial complications associated with EBV, screening for EBV infection in all tonsillitis cases is worthwhile to identify high-risk patients and safety net them accordingly. These patients can then be followed up to check for progression and be monitored for signs and symptoms that may require urgent medical intervention.

Awareness of the impact of EBV infection and its global burden of death should be raised among healthcare staff. It has been noted that EBV, although rare, can lead to several malignancies. As previously mentioned, the incidence of EBV-related malignancies has increased along with the associated mortality. With increased worldwide life expectancy, we expect the EBV death burden to also rise in the future. This warrants serious consideration by healthcare staff in identifying affected cases and ensuring they are managed quickly. This will necessitate early detection of complications and timely interventions to reduce the overall burden.

While our research brought to attention the breaches in healthcare provision when approaching cases with tonsillitis symptoms, it has some limitations. First, the study was based in a single centre, which may limit the generalisation to a wide population. Second, Lincoln City has a high elderly population, which could skew the data given that the prevalence of EBV infection is more common in young adults. Third, this is a retrospective study, limiting our ability to accurately deduce whether those who tested positive developed any complications or received appropriate safety netting. Further studies can utilise our study findings to investigate a wider population and follow-up EBV-positive cases to assess the prognosis and related complications further.

## Conclusions

Tonsillitis is a common presentation in healthcare, with EBV being the leading cause. Epstein-Barr virus is a common infection with high seropositivity worldwide. It has been noted that EBV can be associated with several complications, and those with the infection should be made aware of potential risks and necessary preventive measures.

The study aimed to contribute valuable insights into the prevalence of glandular fever among tonsillitis patients, the significance of conducting glandular fever screenings, and potential connections between EBV infection, liver function abnormalities, and tonsillitis occurrences within the study population. Furthermore, it addressed the gap in healthcare in detecting EBV cases and deduced that there is a probability of a significant number of potential missed EBV cases. Given the widespread EBV infection and increased liability for related issues, we highlight the need to consider involving EBV screening as part of the investigation line in tonsillitis patients.

Such comprehensive analyses and associations sought to advance our understanding of the interplay between tonsillitis, glandular fever, and related physiological implications. This will aid in taking crucial measures to manage and ensure the safety of our patients, possibly influencing future diagnostic and treatment strategies to improve healthcare provision in our population.
